# Melatonin ameliorates the adrenal and pancreatic alterations in streptozotocin-induced diabetic rats: Clinical, biochemical, and descriptive histopathological studies

**DOI:** 10.3389/fvets.2022.1016312

**Published:** 2022-10-21

**Authors:** Atif Abdulwahab A. Oyouni, Osama M. Al-Amer, Fatma Abo Zakaib Ali, Malik A. Altayar, Mohammed M. Jalal, Rayan Salem M. Albalawi, Abdulwahab Ali Abuderman, Khalaf F. Alsharif, Waseem AlZamzami, Ashraf Albrakati, Ehab Kotb Elmahallawy

**Affiliations:** ^1^Department of Biology, Faculty of Sciences, University of Tabuk, Tabuk, Saudi Arabia; ^2^Genome and Biotechnology Unit, Faculty of Sciences, University of Tabuk, Tabuk, Saudi Arabia; ^3^Department of Medical Laboratory Technology, Faculty of Applied Medical Sciences, University of Tabuk, Tabuk, Saudi Arabia; ^4^Department of Pathology and Clinical Pathology, Faculty of Veterinary Medicine, Sohag University, Sohag, Egypt; ^5^Department of Basic Medical Sciences, College of Medicine, Prince Sattam Bin Abdulaziz University, Al-Kharj, Saudi Arabia; ^6^Department of Clinical Laboratory Sciences, College of Applied Medical Sciences, Taif University, Taif, Saudi Arabia; ^7^Department of Human Anatomy, College of Medicine, Taif University, Taif, Saudi Arabia; ^8^Department of Zoonotic Diseases, Faculty of Veterinary Medicine, Sohag University, Sohag, Egypt

**Keywords:** diabetes mellitus, streptozotocin, melatonin, pancreas, adrenal gland

## Abstract

Previous studies have demonstrated the beneficial effects of melatonin in diabetic rats. However, limited studies have been conducted on the potential effects of melatonin on the descriptive histopathological and morphometric findings in different compartments of the adrenal glands in diabetic animal models. In this study, using a streptozotocin (STZ)-induced diabetic rat model, we sought to examine histological alterations in the pancreas and adrenal glands and observe the effect of the administration of melatonin on the histopathology and morphology of the pancreas and the adrenal gland cortex and medulla that are altered by STZ-induced hyperglycemia. Rats were randomly assigned to four different groups: Group I, normal control; Group II, melatonin group (MT) (10 mg/kg/day); Group III, (diabetic STZ group), and Group IV, diabetic (STZ) + melatonin group (MT). Throughout the experiment, the animals' fasting blood sugar levels were measured. Blood was obtained to determine the animals' cumulative blood sugar levels after sacrification. For histological and morphometrical evaluations, the pancreatic and adrenal gland tissues were dissected and processed. Our results showed that diabetic rats receiving melatonin significantly (*P* < 0.05) improved their fasting blood sugar and cumulative blood sugar levels compared to the diabetic group not receiving melatonin. Furthermore, histopathological examinations of the pancreatic and adrenal tissues of the diabetic rats indicated the occurrence of severe histopathological and morphometric changes. Morphometric analysis of the adrenals indicated a significant increase (*P* < 0.05) in the thickness of the cortex zones [zona glomerulosa (ZG), zona fasciculata (ZF), and zona reticularis (ZR)] for the diabetic STZ group compared with other groups, and a significant decrease (*P* < 0.05) in the diameter of the in adrenal gland medullas in the diabetic STZ rats compared to the other groups. Furthermore, treatment with melatonin restored these changes in both the pancreatic and adrenal gland tissues and produced a significant (*P* < 0.05) improvement in the cortex and medulla thickness compared to the untreated diabetic rats. Overall, melatonin significantly reduced the hyperglycemic levels of glucose in diabetic rats and reversed the majority of histopathological alterations in the tissues of the pancreas and adrenals, demonstrating its anti-diabetic activity.

## Introduction

Diabetes mellitus (DM) is a frequently occurring life-threatening metabolic disorder that occurs worldwide. It is characterized by hyperglycemia that is caused by either insulin resistance or declined insulin secretion (1). The incidence of DM has been increasing markedly over the past several decades (2, 3). Previous investigations have demonstrated that DM can lead to various complications, including cardiovascular, neuropathy, retinopathy, nephropathy, and hepatic complications that are known to be the main causes of its associated mortality and morbidity (4, 5). Therefore, it is important that efficient methods for the management and prevention of DM and its complications be identified (6). It is known that induction of experimental diabetes can be caused by chemical destruction or surgical removal of part of the beta cells of the pancreas, by feeding with high-fat and high-sugar diets, and by drugs such as streptozotocin (STZ) and alloxan (7). The pancreatic beta-cell toxin streptozotocin (STZ) causes rapid and irreversible necrosis in these cells. The mechanism of STZ-induced cell injury includes increased reactive oxygen species generation, lipid peroxidation, protein oxidation, and DNA damage that results in cell death (8), producing an insulin deficiency similar to that in human diabetes type I (7).

It should be stressed that the occurrence of DM is closely associated with a dysfunction of the hypothalamic-pituitary-adrenal (HPA) axis (9). A key stress-responsive organ, the adrenal gland is a component of both the HPA axis and the sympatho-adrenomedullary system (10). The adrenal glands are considered to be among the most important endocrine glands and are involved in many biological functions, including developmental, immune, inflammatory, osmoregulatory, and metabolic activities (11). The adrenal cortex produces several steroid hormones The three outer layers of the endocrine gland—the zona glomerulosa (ZG), the zona fasciculata (ZF), and the zona reticularis (ZR)—as well as the inner medulla make up this structure. Numerous steroid hormones that are produced by the adrenal cortex have an impact on metabolism, stress response, and water and electrolyte equilibrium. Previous studies examining the relationship between diabetes and adrenal gland morphology found that patients with diabetes have larger adrenals (9). Polydipsia, polyphagia, and polyuria, which are DM symptoms, have been linked to the adrenal gland (12). All of the body's major organs, including the heart, kidney, bones, muscles, and nervous system, are directly impacted by adrenal hormones, including the metabolic metabolism of carbohydrates, proteins, and lipids (11).

Melatonin (*N*-acetyl-5-methoxytryptamine), an endocrine substance produced from tryptophan, is synthetized primarily by the pineal gland and locally by numerous other tissue cells (13, 14). Investigations on the effects of pineal hormones on insulin secretion, carbohydrate metabolism, and blood glucose levels have been conducted (15, 16). Diabetes mellitus (DM) is associated with reduced serum concentrations of melatonin in diabetic Goto-Kakizaki rats, despite increased insulin levels in these animals (17). Furthermore, melatonin exhibits biological characteristics such as an anti-aging (18), antioxidant, anti-inflammatory (19), and antiparasitic properties (13, 20). Thus, among the stressors, oxidative stress might exist, and that influences the regulation of the HPA axis (21). Therefore, melatonin proves to be beneficial in those cases, as it is a well-known free radical scavenger, antioxidant and antiapoptotic agent (22). Melatonin exerts its functions at all levels, allowing cells to withstand the damage normally inflicted by radicals and radical-related creations (23). There is a broad literature regarding the assessment of adrenal hormones biochemically in patients with DM. However, the interactions between the adrenal cortex and medulla, as well as the structural alterations in adrenal cells' histopathology, especially as they relate to STZ-induced DM, have not been well studied. Furthermore, little information is available on the histopathological changes that occur in the pancreas and adrenal glands in diabetic rats following melatonin administration. Therefore, there is an urgent need for the development of novel drug targets, including natural drugs, with no side effects. In the present study, our goal is to investigate the histopathological deviations that occur in the pancreas and adrenal glands in a streptozotocin (STZ)-induced DM rat model, which can shed light on the pathophysiology of the adrenal glands as the STZ-induced DM progresses. In addition, we planned to observe the effect of melatonin on the morphology of the adrenal cortex and medulla that are altered by STZ-induced hyperglycemia, which would provide basic knowledge regarding DM and its treatment.

## Materials and methods

### Ethical considerations

Animal handling and rights were conducted in accordance with the ethical committee guidelines of the Faculty of Veterinary Medicine, Sohag University. The ethical approval number is Sohag Vet/2021-1.

### Materials

#### Drugs, chemicals, and oils

Streptozotocin powder, trisodium citrate dihydrate, and citric acid monohydrate were purchased from the Sigma-Aldrich Company (St. Louis, MO, USA). Melatonin (*N*-acetyl-5-methoxytryptamine) was purchased from FAGRON (Cat# 420 33457-24, Fagron, Nazareth, Belgium).

#### Animals

Forty mature male Wister albino rats, at 6 weeks of age with average body weights of 165–200 g, were purchased from the lab animal facility of the Faculty of Medicine, Sohag University, Sohag, Egypt. Animals were housed in clean and pathogen-free stainless-steel cages (five rats in each cage) with a 12 h light/12 h dark cycle, at 23 ± 2°C, and 50%−55% humidity. Rats were fed a standard pellet diet and water *ad libitum* throughout the experimental period. The bedding was changed continuously to ensure a clean environment. They were kept under these conditions for 1 week to acclimatize them before starting the experiment.

### Experimental approach and design

To avoid using animals that were already infected with parasites, samples from the sediment and the supernatant after centrifugation were tested separately for the presence of parasitic eggs and/or larvae as part of the weekly fecal sample analysis. The rats were then divided into four groups of 10 each: Group I, a normal control group (*n* = 10) that was given regular rat food and water; Group II, the melatonin group (MT; *n* = 10) received an IP injection of melatonin, 10 mg/kg/day, for 4 weeks. The melatonin was dissolved in DMSO (1%, w/v) prior to injection (24–28). Group III, the diabetes STZ group (*n* = 10), were fasted the previous night for 12 h before inducing diabetes. Group IV, the diabetes (STZ) + melatonin group (MT; *n* = 10), were administrated melatonin IP (the same as for Group II) after treatment with STZ (the same as for the diabetic control positive Group III). For the induction of diabetes, a single IP injection of freshly prepared streptozotocin (45 mg/kg body weight, in 0.1 M cold citrate buffer, pH 4.5) was administered (25, 29). The animals were allowed free access to food, water, and a 15% solution of glucose overnight after the STZ injections to prevent hypoglycemic shock. The blood glucose levels were examined 3–6 days after the STZ injections (24, 30–32). Seventy-two hours after the injection of STZ, evidence of diabetes was observed by the occurrence of polyuria and polydipsia, and by measuring blood glucose levels from a blood sample taken from the tail vein, using a glucometer (On Call Plus, ACON Laboratories, Germany). Only those rats that received STZ and had blood glucose levels of 250 mg/dl or greater were considered diabetic and chosen for the study. All rats used for diabetic induction successfully developed diabetes following the STZ injection. Melatonin was administrated at the previously mentioned dose to the rats in the melatonin diabetic groups. Twelve hours after the final administration of melatonin, all the rats were sacrificed, and blood and tissue samples were taken.

### Methods

#### Measurements of animal body weight

The body weights of the animals were measured before the start of the experiment and at the time of sacrifice.

#### Sample collection

##### Blood samples for the estimation of fasting blood sugar levels

Following blood collection from animal tail veins that had been disinfected with 10% alcohol during the experiment, fasting blood glucose levels were assessed. A tail prick was used to get a blood sample, which was then put on a test strip and put into a calibrated glucose meter (On Call Plus Glucometer, ACON Laboratories, Germany) as described by Airaodion et al. (32) and Togashi et al. (33). The results for fasting blood sugar levels were obtained after 5 s and were expressed as mg/dl.

###### Total blood samples

At the end of the experiment, the rats were sacrificed, and individual blood samples from each group were collected in clean, dry tubes containing EDTA as an anticoagulant for the assessment of glycosylated hemoglobin. (HbA1C) (34).

### Biochemical analysis

#### Measuring of fasting blood glucose levels

Fasting blood glucose levels were determined during the experiment as described previously in the experimental design (33).

#### Estimation of cumulative blood sugar (glycosylated hemoglobin)

An Arkray Automatic Glycohemoglobin Analyzer, ADAMS A1c HA-8190V, a completely automated Glycohemoglobin (HbA1c) analyzer that utilizes High Performance Liquid Chromatography, was used to determine cumulative blood sugar levels. The HA-8190V automatically distinguishes and detects various forms of hemoglobin (34).

### Histopathological examinations

Animals were sacrificed at the end of the experiment, and tissue samples from the pancreas and the adrenal glands were taken. These samples were then dissected, fixed immediately in 10% formalin for 24 h, dehydrated using a graded alcohol series, cleaned in xylene, and then embedded in paraffin. Sectioned tissues, 3 μm thick, were stained with hematoxylin and eosin (H&E) (35), for histopathological examination. All sections were examined using an OLYMPUS CX43 light microscope and photographed using an OLYMPUS SC52 camera from the microscopy unit of the Faculty of Veterinary Medicine, Sohag University, Department of Pathology and Clinical Pathology.

### Histomorphometric assessments

Organ histological analyses were conducted at this stage by assigning a score that depended on the degree of damage observed in each group in the tissue under examination: 0 = no lesions; 1 = minimal (1–10); 2 = mild (10%−35%); 3 = moderate, (36%−50%); and 4 = severe (>50%) which were modified based on previous assessments (36–39). ImageJ version 1.48 software (NIH) was used for measuring the surface area of adrenal glands, the individual heights of the zona granulosa (ZG), zona fasciculata (ZF), and zona reticulata (ZR), and the height of the medulla (M) in H&E stained sections. A total of five samples from each group (five replicates) were included from each paraffin block, then five separate, non-overlapping sections were selected and inspected using low-power fields (×200) (40). Morphometry was performed in the Image Analysis Unit, Department of Pathology and Clinical Pathology, Faculty of Veterinary Medicine, Sohag University.

### Statistical analysis

The data were expressed as the mean ± standard deviation (SD), The measurements obtained from the experimental groups were estimated statistically using GraphPad Prism, version 5 (San Diego, California, USA) using a one-way ANOVA with Tukey's *post hoc* multiple comparison tests; *P*-values were used to compare and define the statistical significance between groups (41–43). A paired *t*-test was used to compare the body weight as well as fasting blood glucose levels between pre- and post-treatment. A difference was considered significant at *P* < 0.05.

## Results

### Clinical and biochemical profiles

As a consequence of STZ-induced diabetes, rats developed type 1 DM within 72 h and exhibited characteristic DM symptoms such as polyphagia, polydipsia, polyuria, and unexplained and significant (*P* ≤ 0.05) weight loss at the end of the experimental trial (Table 1). By comparing the STZ diabetic group to the STZ + MT-treated group and the control group, it was clear that the lowered body weights in the STZ diabetic group were significant (*P* ≤ 0.05; Table 1). The statistical analysis of fasting blood sugar during the first and fourth week of the experiment revealed that both groups, including the diabetic STZ group and the diabetic STZ + MT-treated group, had significantly (*P* ≤ 0.05) increased blood glucose levels compared to those in the control negative group. During the fourth week, the mean value for fasting blood sugar in the diabetic STZ + MT-treated group decreased significantly (*P* ≤ 0.05) compared to the control positive group; however, a difference was observed between the control negative and MT control groups (Table 2). When compared to the control negative and MT control groups, the diabetic STZ group's cumulative blood sugar (HbA1c) levels were the highest and was significantly (*P* ≤ 0.05) higher, indicating that the rats in this group had diabetes. In the diabetic STZ + MT-treated group, the mean value of the level of cumulative blood sugar was significantly (*P* ≤ 0.05) higher when compared to the control and MT control groups, but it was also significantly lower compared to the diabetic STZ group (Table 3).

**Table 1:** Comparison of body weights in experimental groups in the STZ DM model.

**Groups**	**Body weight (g.)**				***P*-values of paired *t*-test**
	**(Mean** ±**SD)**				
	**Initial body weight** ** (before the experiment)**	**Final body weight** **(before sacrifice)**			
Control	177.1 ± 15.41	207.6 ± 19.50			0.083
MT control	166.5 ± 6.028	204.6 ±10.11^ns^	^***^	STZ vs. STZ + MT^*^	0.352
Diabetic (STZ)	192.4 ± 12.53	144.4 ± 13.48^***^		MT vs. STZ + MT (ns)	0.0006
Diabetic STZ + MT-treated	190.3 ± 24.12	176.4 ± 24.46^*^			0.351

Values are expressed as means ± SD.

Significant differences vs. the control group are marked by different asterisks through one-way ANOVA with Tukey's *post hoc* test:

* *P* ≤ 0.05,

*** *P* ≤ 0.001, ns: Non significant.

The *P*-values of the paired t-test were 0.083, 0.352, 0.0006, and 0.351 for Control, MT control, Diabetic (STZ), and Diabetic STZ + MT-treated respectively. For each group (n = 10).

**Table 2:** Comparison of fasting blood glucose levels in the experimental groups.

**Groups**	**Fasted blood glucose level**				***P*-values of paired *t*-test**
	**(Mean** ±**SD)**				
	**First week after** ** diabetic induction**	**Fourth week after diabetic induction** **(Before sacrifice)**			
Control	86.00 ± 5.888	66.20 ± 7.190			0.123
MT control	105.0 ± 5.196^ns^	66.25 ± 3.775^ns^	STZ vs. MT control ^***^	STZ vs. STZ + MT ^**^	0.425
Diabetic (STZ)	420.3 ± 52.67^***^	362.0 ± 123.1^***^		MT vs. STZ + MT	0.0042
Diabetic STZ + MT-treated	395.3 ± 36.47^***^	196.7 ± 98.54^ns^		(ns)	0.0002

Values are expressed as means ± SD.

Significant differences vs. the control group are marked by different asterisks through one-way ANOVA with Tukey's *post hoc* test:

** *P* ≤ 0.01,

*** *P* ≤ 0.001, ns: Non significant.

The *P*-values of the paired t-test were 0.123, 0.425, 0.0042, and 0.0002 for Control, MT control, Diabetic (STZ), and Diabetic STZ + MT-treated respectively. For each group (n = 10).

**Table 3:** Comparison of cumulative blood sugar values (HbA1C) between experimental groups in the STZ DM model.

**Groups**	**Glycosylated hemoglobin (HbA1C)**		
	**(Mean** ±**SD)**		
Control	74.32 ± 5.584		
MT control	72.68 ± 8.266^ns^	STZ vs. MT control ^***^	STZ vs. STZ + MT ^***^
Diabetic (STZ)	212.4 ± 12.06^***^		
Diabetic STZ + MT-treated	123.3 ± 25.67^**^		MT vs. STZ + MT (^**^)

Values are expressed as means ± SD.

Significant differences vs. the control group are marked by different asterisks through one-way ANOVA with Tukey's *post hoc* test:

** *P* ≤ 0.01,

*** *P* ≤ 0.001).

For each group (n = 10).

### Histopathological assessments

#### Pancreatic tissue

**Figure 1:**
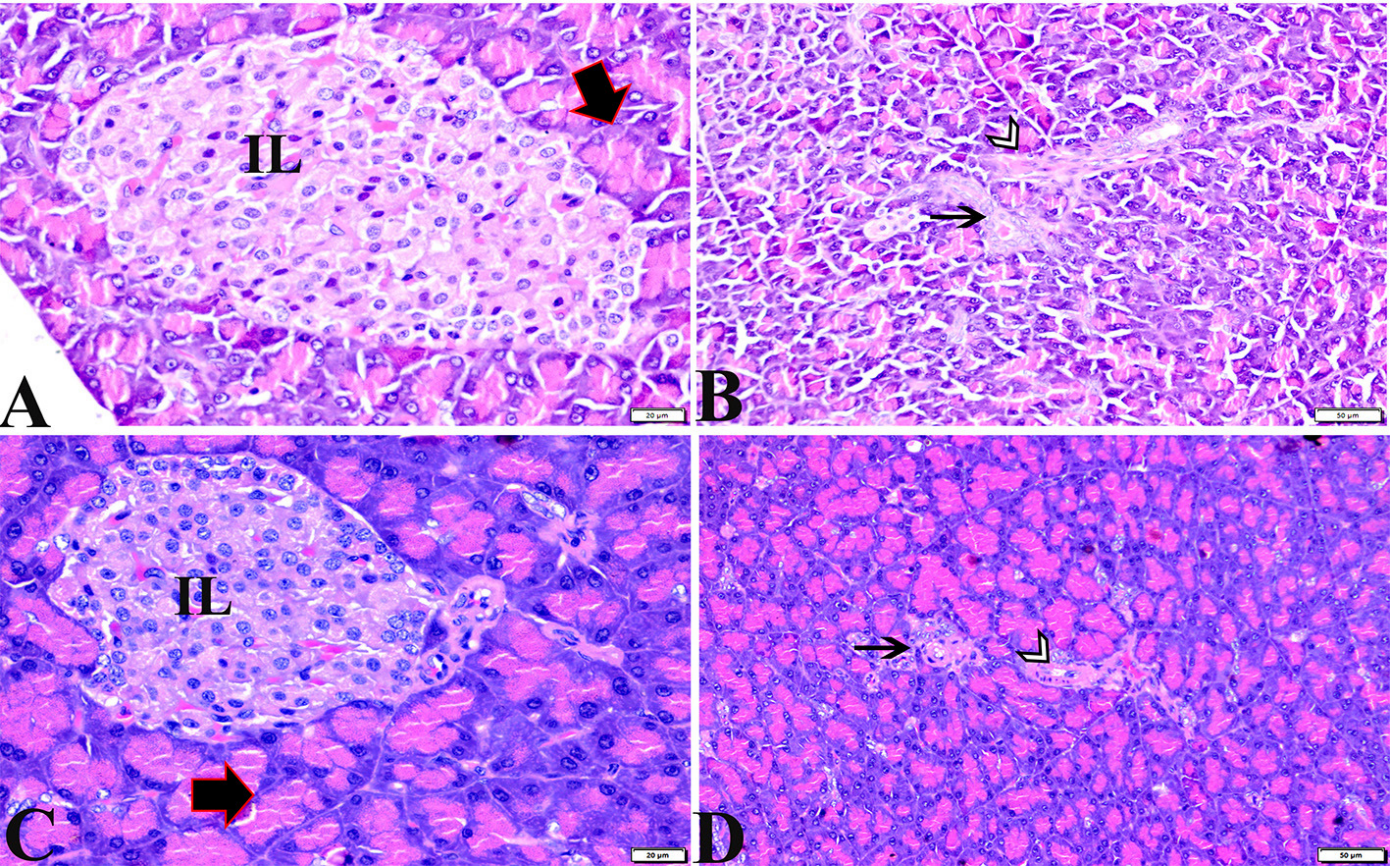
Photomicrographs of pancreatic sections from the control negative group **(A,B)** and the MT control group **(C,D)** demonstrating normal pancreatic structure and architecture in the form of normal-sized islets of Langerhans and normal density of islet cells, normal exocrine acinar cells (thick arrows), a normal intralobular duct (arrowheads), and normal blood vessels (thin arrows). H&E stained. The bar size is indicated under each picture.

**Figure 2:**
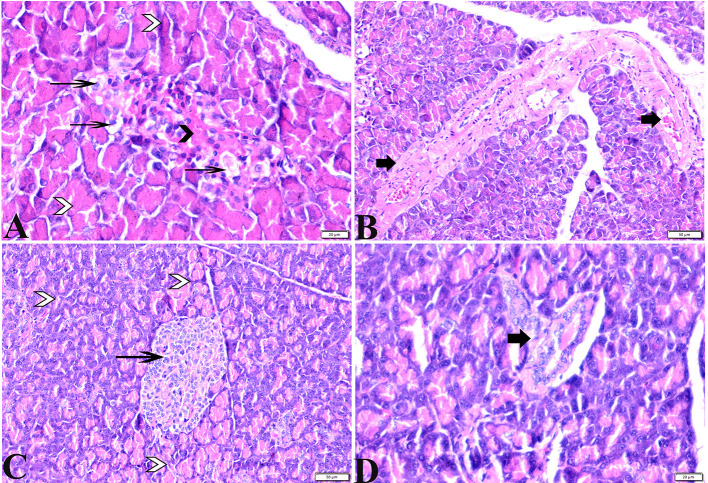
Photomicrographs of pancreatic sections from **(A,B)** of the diabetic STZ group showing **(A)** islets of Langerhans that experienced depletion and necrobiotic changes in its constituent cells, vacuolated degeneration in most of the cells (thin arrows), and capillary hemorrhage (black arrowheads). The exocrine acinar cells exhibit degeneration and dissociation (white arrowheads). **(B)** severely dilated and congested blood vessels with thick vascular walls (arrows). **(C,D)** diabetic STZ + MT-treated group showing: **(C)** well-defined islets of Langerhans with proliferated cell populations (arrow) in between normal pyramidal acidophilic pancreatic acini (white arrowheads). **(D)** a normal vascular structure (thick arrow). H&E stained. The bar size is indicated under each picture.

The histopathological examinations of pancreatic tissue sections from the various experimental groups revealed the same normal histological structures in both the control negative and MT-treated rats and reflected the typical architecture of the pancreatic endocrine and exocrine parenchymal structures. Large pale oval regions with a distinct contour between the exocrine acini were visible as the endocrine islets of Langerhans. They were composed of numerous microscopic pale β cells and a few spherical, large, acidophilic cells encircling tiny blood capillaries (Figures 1A,C), in between numerous pyramidal acinar cells (Figures 1B,D). The tissue sections from the diabetic STZ rats without any treatment (control positive group) revealed a number of pathological findings, including distorted pancreatic lobules and a necrobiotic change in cells of the islets of Langerhans in which the cytoplasm was homogenous and coagulated or vacuolated, the cytoplasm exhibited lysis, the nucleus was pyknotic, and other regions were lysed or absent, and the destroyed cells were replaced by fatty tissue (Figure 2A). This group's exocrine parenchymal region exhibited atrophy, degeneration, and dissociation of a few exocrine acini (Figure 2A). Generally, the vascular system of the pancreatic tissue from this group (control positive group) revealed severe congestion with very thick walls (Figure 2B). Melatonin (MT) was concurrently administered to diabetic STZ rats in Group IV, which alleviated the pathological features that were noted in the diabetic STZ rats in Group III, where many normal pancreatic lobules with thin interlobular septa were found. The exocrine parenchyma had multiple pancreatic acini of normal size, and the islets of Langerhans and cells showed enhanced morphological appearance (Figure 2C). Moreover, the blood vessels were normal in structure (Figure 2D).

#### Adrenal glands

**Figure 3:**
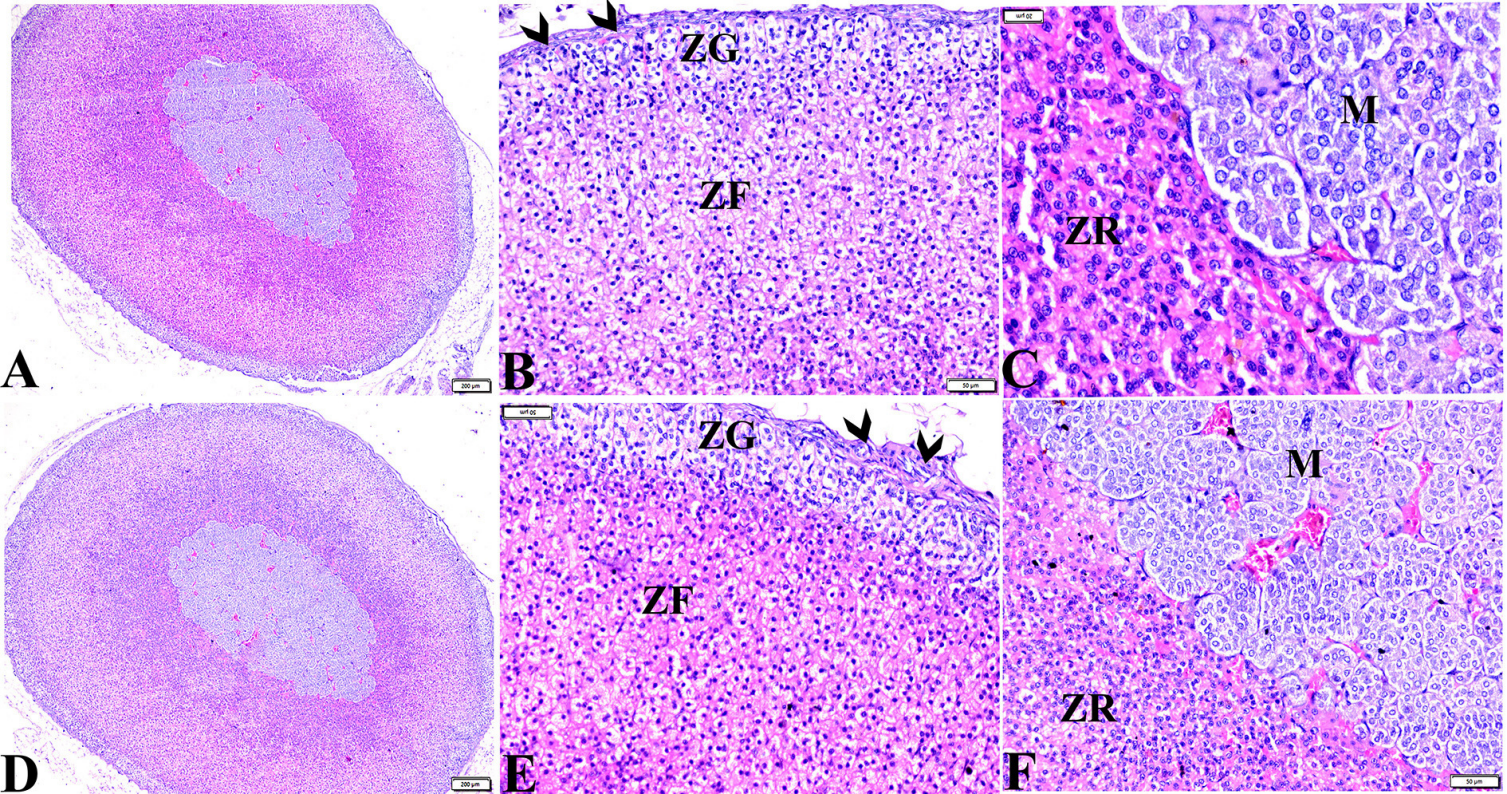
Photomicrographs of adrenal gland sections from **(A–C)** control negative group and MT control group **(D–F)** indicate thin connective tissue capsules (arrowheads). A normal adrenal cortical structure comprised of ZG, ZF, and ZR cells, and a normal adrenal medulla. H&E stained. The bar size is indicated under each picture.

**Figure 4:**
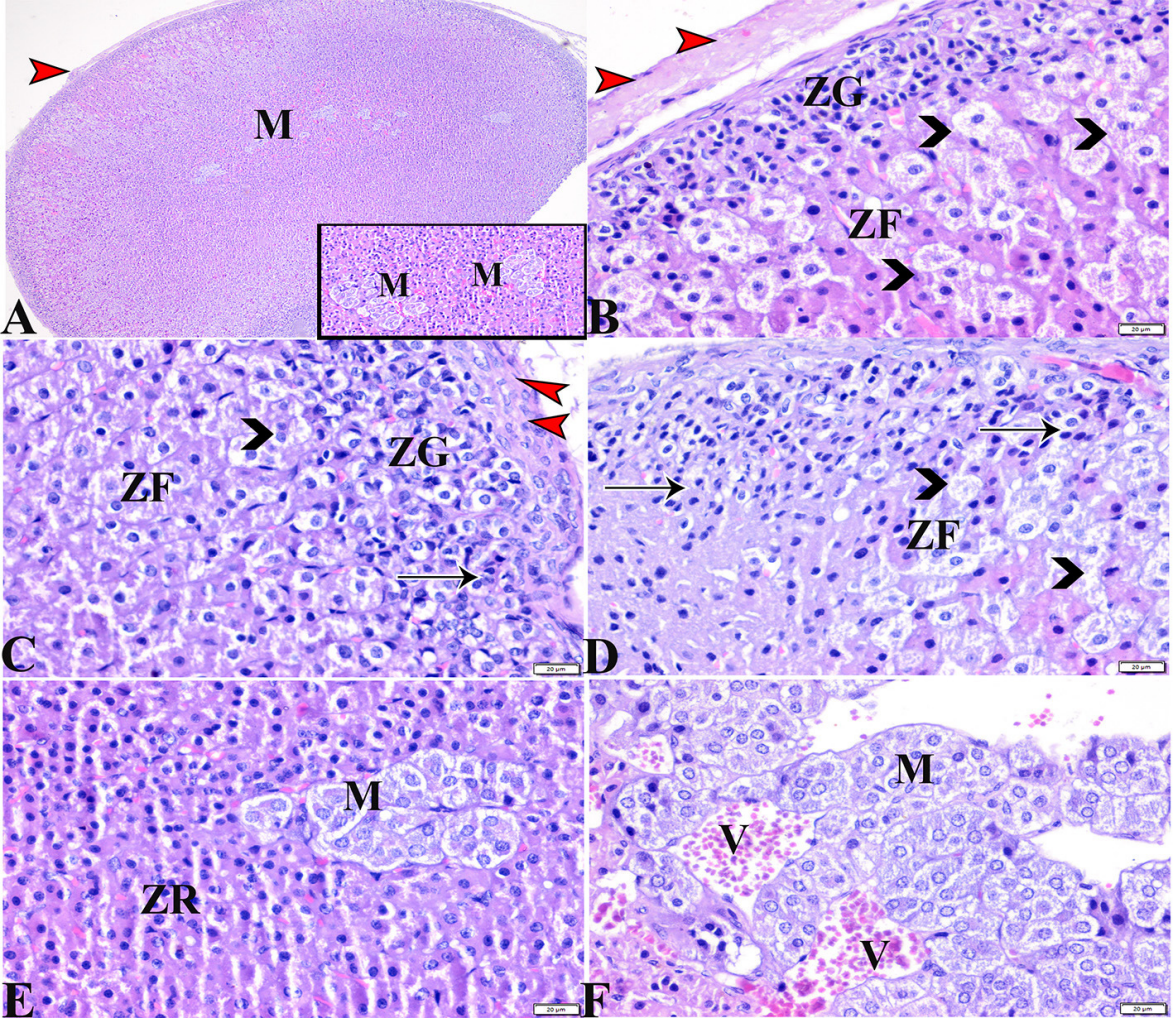
Photomicrographs of adrenal gland sections from the diabetic STZ group **(A–F)** showing: **(A–C)** Thick connective tissue capsules (red arrowheads), distortion in the adrenal cortical structures; Zona granulose (ZG) and Zona fasciculate (ZF), in addition to adrenal medulla (M) [**(A)**, selected square]. **(B–D)** Hyperplasia in the ZG cell layer [**(B,C)**, ZG], with pyknotic cellular nuclei [**(C,D)**, thin arrows], hyperplasia and vacuolation of the ZF cellular layer, loss of its radial arrangement and some cells contain karyolysis nuclei [**(B–D)**, arrowheads]. **(E)** Distorted ZR cells (ZR). **(E,F)** Atrophied adrenal medulla with vacuolated chromaffin cells (M). **(F)** The medullary veins are severely dilated and engorged with blood (V). H&E stain. The bar size is indicated under each picture.

Microscopic examinations of tissue sections from the adrenal glands from the various experimental groups revealed the same histological structures in both the control negative and MT-treated rats which possessed the typical histological size and morphological architecture of adrenal glands encased in a connective tissue capsule that extends septae into the substance of the gland (Figures 3A,C). Distinctive features of adrenal partitioning into the cortex and medulla included the following: the cortex consisted of three concentric zones with the outermost thin zona glomerulosa, the middle thick zona fasciculata, and the inner thin zona reticularis (Figures 3B,E). In the adrenal medulla, the most abundant cells were the columnar basophilic granular chromaffin cells which were arranged in clusters, usually around the medullary veins (Figures 3C,F). Sections of the STZ diabetic rat adrenal glands exhibited distortion in their architecture, such as thickening in the connective tissue capsule, hyperplasia, hypertrophy in the cortical structures, hyperplasia in the zona glomerulosa cell layer with pyknotic cellular nuclei, and hyperplasia and vacuolation of the zona fasciculate cellular layer with a loss of its radial arrangement. Some cells contained karyolitic nuclei, and the zona reticularis cells were also distorted. The adrenal medulla chromaffin cells were atrophied, and vacuolated, and most lost their cytoplasmic granules. The capillary cortex and medullary veins were severely dilated and engorged with blood (Figures 4A–F).

**Figure 5:**
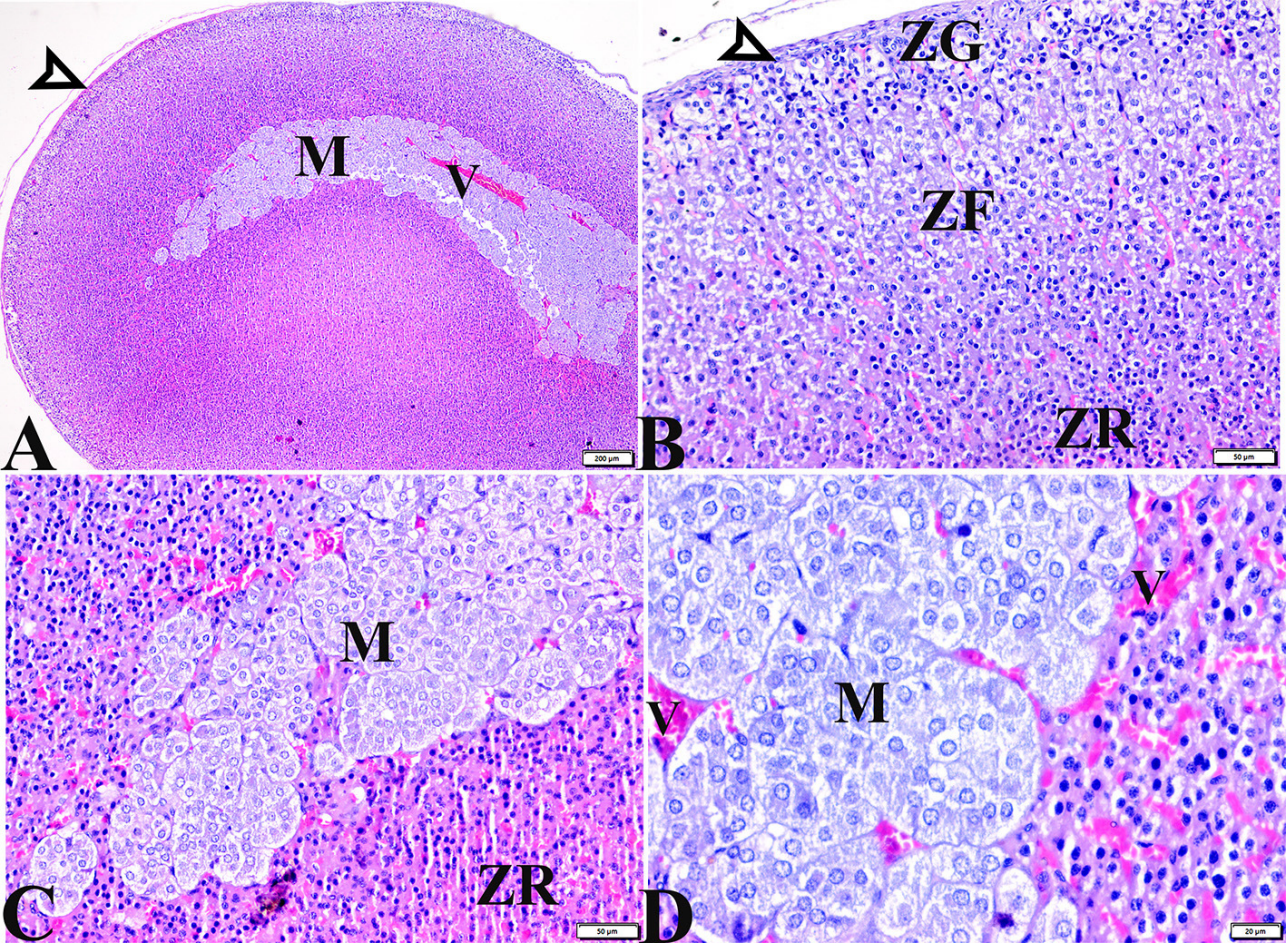
Photomicrographs of adrenal gland sections from the diabetic STZ + MT-treated group showing adrenal tissue regenerations that contain **(A,B)** Normal thin connective tissue capsule (white arrowheads). **(B)** A remarkable improvement in cortical zonal partitions, a mild vacuolated ZG cell layer (ZG), mild vacuolation of the ZF cellular layer with normal arrangement (ZF), and a normal ZR cell layer (ZR). **(A,C,D)** Normal adrenal medulla basophilic chromaffin cells with granular cytoplasm (M). **(D)** Mild congestion in capillary medullary veins (V). H&E stained. The bar size is indicated under each picture.

The histopathological examination of adrenal tissue sections from diabetic STZ + MT-treated rats revealed a marked improvement in the adrenal histological structure and a remarkable restoration to normal architecture in the zona glomerulosa, the zona fasciculate, and the zona reticularis cell layers, except for some vacuolar degeneration. Animals in this group also contained normal adrenal medulla basophilic chromaffin cells with granular cytoplasm, while capillary medullary veins exhibited mild congestion (Figures 5A–D).

### Quantitative and semiquantitative histomorphometric studies

#### Scoring of pancreatic tissue injury

**Figure 6:**
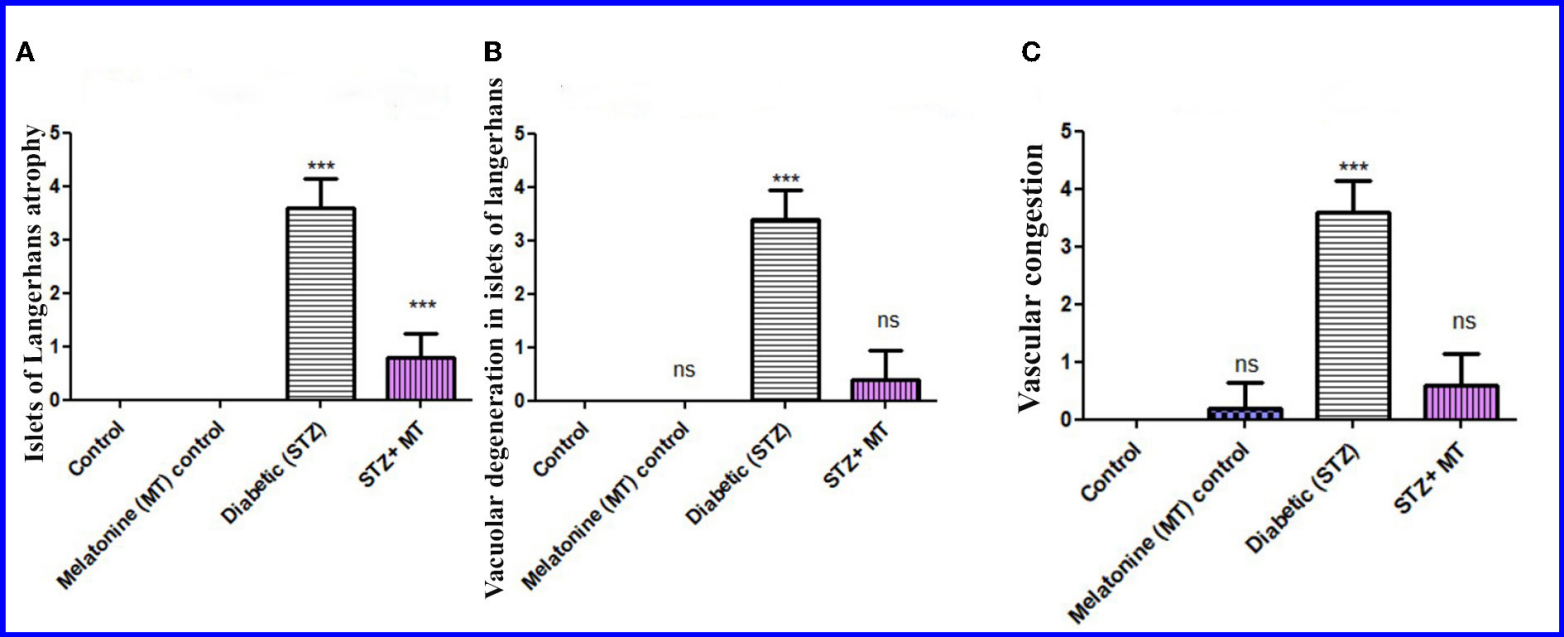
Histomorphometry graph showing semiquantitative measurements of pancreatic tissue sections among the experimental groups. **(A)** Atrophy of the islets of Langerhans, **(B)** Vacuolar degeneration in the islets of Langerhans, and **(C)** Vascular congestion. Values are expressed as means ± SD. Significant differences vs. the control group are marked by different asterisks through one-way ANOVA with Tukey's *post hoc* test: ns, non-significant; ***P ≤ 0.001.

Compared to other groups, the diabetic STZ group's pancreatic histomorphometric results exhibited a significant difference (*P* < 0.05) in the type of cell damage, which was manifested by atrophy of the islets of Langerhans (Figure 6A), vacuolar degeneration in the islets cells (Figure 6B), and vascular congestion (Figure 6C). Alternatively, diabetic rats treated with melatonin exhibited no significant change (*P* < 0.05) compared with control groups.

#### Adrenal gland damage scoring

##### Size of adrenal gland

**Figure 7:**
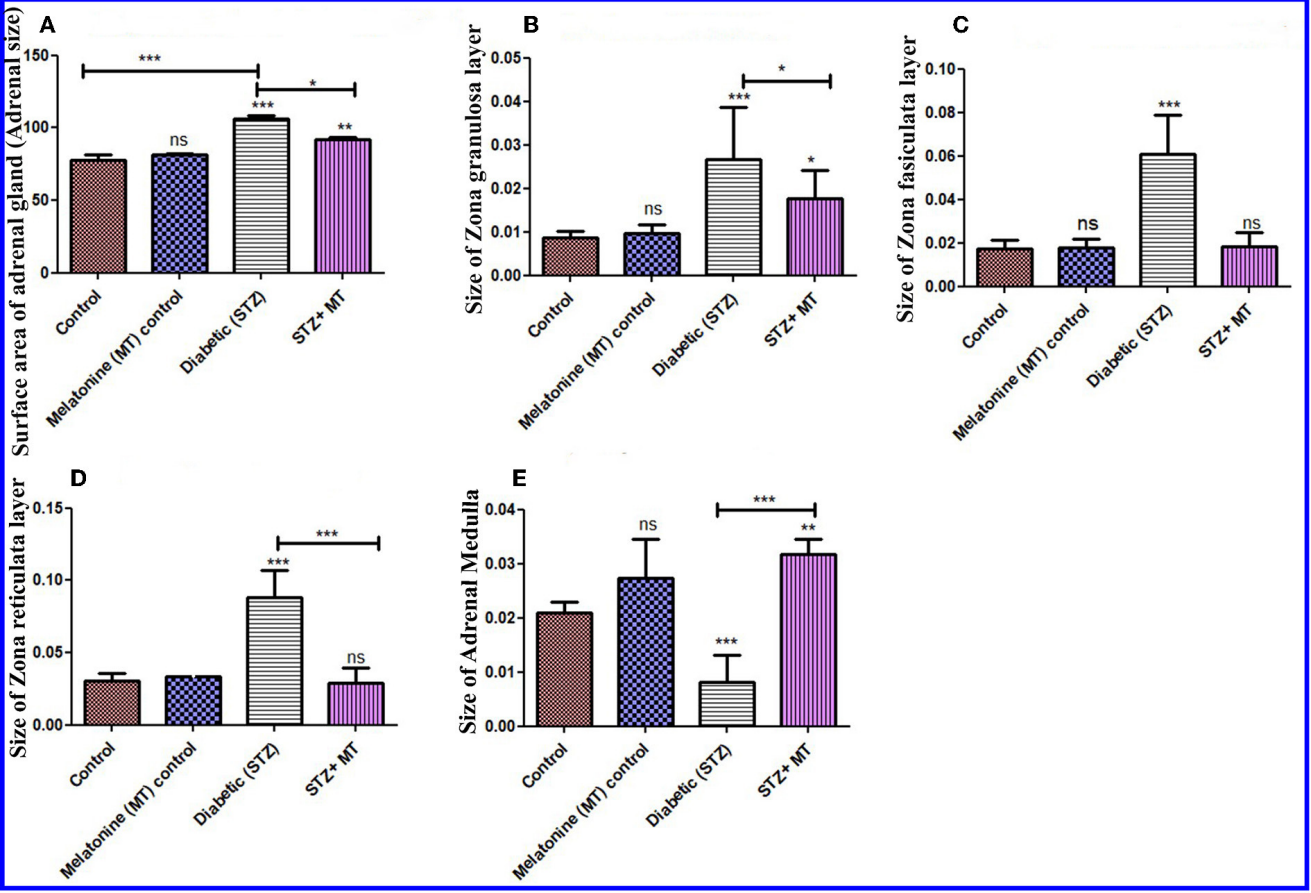
Histomorphometry graph showing quantitative measurements of adrenal tissue sections among the experimental groups. **(A)** Surface area of adrenal glands (size), **(B)** Size of the ZG layer, **(C)** size of the ZF layer, **(D)** size of the ZR layer, and **(E)** size of the adrenal medulla. Data are expressed as means ± standard deviations. Significant differences vs. the control group are marked by different asterisks through one-way ANOVA with Tukey's *post hoc* test: *P ≤ 0.05, **P ≤ 0.01, ***P ≤ 0.001.

Histomorphometric measurements of the adrenal surface areas for the diabetic STZ group and the diabetic STZ + MT-treated group indicated that both were significantly increased (*P* < 0.05) compared to the other groups. The adrenal size was significantly increased (*P* < 0.05) in the diabetic STZ rats compared to the diabetic STZ + MT-treated group (Figure 7A).

##### Histomorphometry for thickness of cortex and medulla

There was a significant increase (*P* < 0.05) in the thickness of the cortex zones (ZG, ZF, and ZR) for the diabetic STZ group compared to the other groups, and a significant decrease (*P* < 0.05) in the diameter of the medulla in the adrenal gland of diabetic STZ rats compared to the control and other groups. The diabetic rats treated with melatonin exhibited a significant (*P* < 0.05) improvement in the total thickness of the cortex and medulla compared to the untreated diabetic rats (Figures 7B–E).

## Discussion

Diabetes mellitus causes a variety of complications and affects many body organs, which are considered the principal triggers of its morbidity and mortality (4, 5).

The induction of experimental diabetes with streptozotocin caused rapid and irreversible pancreatic β-cell injury which involved the production of a massive generation of reactive oxygen species, lipid peroxidation, protein oxidation, and DNA damage resulting in the death of β-cells (8), all associated with insulin deficiency (7). As mentioned in previous studies, DM instigated malfunction of the HPA axis and developed an alteration of the adrenal glands in Wistar rats (9).

Melatonin's impact on the pancreatic tissues and adrenal glands in STZ-induced diabetes was examined in the current study. *N*-acetyl-5-methoxytryptamine, often known as melatonin, is a secretory byproduct of the pineal gland and other tissue cells that has potent scavenging activity for hydroxyl and peroxyl radicals, in addition to regulating the activity of antioxidant enzymes which is one of its functions (44, 45). Several previous studies have looked into the outcomes of administrating of melatonin in diabetic rats (15, 16). In the current work, rats developed glucosuria, hyperglycemia, and unexpected significant (*P* < 0.05) weight loss after diabetic induction compared to other groups (Table 1), as has been described in previous studies (46, 47). Melatonin in our study was unable to increase the body weight of the STZ-treated rats compared to control animals. However, the findings of body weight alterations were significant (*P* < 0.05) compared to the diabetic STZ-untreated rats. Parallel findings have been reported previously (45, 48). The sympathetic nervous system is thought to be implicated in this process by amplifying its effects on the brown and white adipose tissues, which cause them to mobilize and expend energy (16, 49).

In the research reported here, the administration of melatonin (10 mg/kg) in diabetic STZ rats induced a reduction in fasting blood glucose levels, which were equivalent to the healthy control levels (Table 2). Statistical analysis of fasting blood glucose levels after the first week of the experiment revealed that both diabetic STZ and STZ + MT-treated groups had significantly (*P* ≤ 0.05) elevated blood glucose levels compared to those of the control groups. After the fourth week, and before the animals were sacrificed, the blood glucose levels declined significantly (*P* ≤ 0.05) in the diabetic group treated with melatonin compared to the diabetic STZ-untreated group. Our results are in agreement with those of Doosti-Irani et al. (50) who documented that melatonin helps to maintain glycemic homeostasis by improving insulin sensitivity and lowering fasting blood sugar levels. As shown in Table 3, the uppermost level of HbA1c was monitored in the diabetic STZ group which was significantly (*P* ≤ 0.05) higher than the control groups. However, the diabetic STZ + MT-treated group expressed significantly (*P* ≤ 0.05) upraised HbA1c levels compared to the control and MT control groups, indicating that the treatment with melatonin had no discernible impact on the STZ-induced diabetic rats' plasma glucose levels or the emergence of hyperglycemia. These findings are in agreement with the results of other researchers (49, 51). However, the HbA1c levels in diabetic STZ rats treated with melatonin were significantly lower compared to the diabetic STZ-untreated group. Our results agree with Sartori et al. (52) who also observed that melatonin therapy improved glucose tolerance. After 5 months of use, melatonin reduced HbA1c levels (53), and enhanced glycemic control in diabetic patients (54). In addition, Shima et al. (55) had stated in their study that hyperglycemia instigated by the intracerebroventricular injection of 2-deoxy-D-glucose in rats was suppressed by melatonin. They reported that melatonin injections suppressed blood glucose levels, possibly through a brain site. A previous study (56) also stated that melatonin may decrease blood glucose levels through its role on catecholaminergic responses.

Some islets of Langerhans became severely atrophied in this study, and the surrounding exocrine tissue displayed degenerative changes. Some islets cells underwent necrobiotic changes, and the damaged cells occasionally were replaced by fatty tissue. The vascular system of the pancreas displayed considerable congestion, according to prior research (57, 58). These results may be due to DNA destruction caused by streptozotocin and ROS generation in small amounts with a decrease in antioxidant enzymes (59). A previous study (60) showed that in patients with poorly managed type II diabetes and metformin treatment, there was a greater cellular response after receiving 10 mg of melatonin. Another study (61) found that in obese people with acanthosis nigricans, treatment with 3 mg of melatonin per day, for 12 weeks, increased insulin sensitivity and decreased inflammation. Furthermore, the administration of melatonin diminished the levels of cytokines and immunoglobulins that consequently reduced pancreatic inflammation and allowed the regeneration of β cells (62). This was noticeable in the pancreatic tissue sections of the MT animals, where the size and structure of the islets recovered to some extent compared to the untreated diabetic group.

It should be stressed that the adrenal glands are involved in many biological functions including metabolic, osmoregulatory, immunological, inflammatory, and developmental processes (11). All the body's major organs, including the heart, kidney, bones, muscles, and nervous system, are directly impacted by the changes that adrenal hormones cause in the metabolism of carbohydrates, proteins, and lipids (11). Additionally, the adrenal gland is critical in escalating DM symptoms (63). In this respect, several studies have illustrated that diabetes causes severe degeneration within the cells of the zona glomerulosa and the zona fasciculate of the adrenal cortex (9, 64). The HPA axis was found to be hyperactive in the presence of hyperglycemia, which raises plasma levels of both cortisol and corticosterone (65) and high blood glucose levels consequently happened. The histology of diabetic ZR is still unknown, though it has been shown that the adrenal cortex and medulla have medullary cellular interactions at various levels, with cortical hormones promoting medullary hormonal synthesis and vice versa (66). Both adrenal hypertrophy and hyperplasia have been reported in STZ-induced diabetes (67). These reported findings in previous studies are in agreement with our present observations in relation to the histopathological effects of diabetes induced by STZ on cellular structures, the morphology of adrenal glands, thickening in the connective tissue capsule, distortion, hyperplasia, hypertrophy, vacuolation, and necrobiotic changes in the cortical structures of ZG, ZF, and ZR cells.

Furthermore, our present study revealed that adrenal medulla chromaffin cells were also atrophied and vacuolated and most of them lost their cytoplasmic granules. In addition, vascular congestion and dilatation were also observed. In the present study, the diabetic rats treated with melatonin exhibited a marked improvement in adrenal histological structures, and a remarkable restoration to most of its normal architecture in the ZG, ZF, and ZR cell layers, except for some vacuolar degeneration. In addition, the adrenal medulla contained normal basophilic chromaffin cells with granular cytoplasm and mild vascular congestion. When compared to diabetic rats that were left untreated, melatonin also dramatically decreased medulla overall thickness and was able to restore the morphology and hypertrophy of the cells in the adrenal cortex (*P* < 0.05). These results are consistent with previous research on the adrenal cortex (68).

## Conclusions

The present study demonstrated that histopathological and morphological changes of the adrenal gland cortex compartments and medullary cellular structure occur in diabetic rats, and a restorative effect in these tissues happen following treatment with melatonin. In addition, the present study established the greatest effect of melatonin in restoring pancreatic cellular structures and adrenal cortex partitions ZG, ZF, and ZR, and for the first time demonstrated its effect in restoring adrenal medulla cellular structure and morphology in diabetic STZ rats. These findings will be useful in further pathological studies on controlling diabetic complications in other body organs.

## Data availability statement

The original contributions presented in the study are included in the article/supplementary material, further inquiries can be directed to the corresponding authors.

## Ethics statement

The animal study was reviewed and approved by the Research, Publication, and Ethics Committee of the Faculty of Veterinary Medicine, Sohag University, Egypt, which complies with all relevant Egyptian legislations in publication and research. The Institutional Review Board Number is Sohag Vet/2021-1.

## Author contributions

AO, OA-A, FA, MA, MJ, RA, AAA, KA, WA, AA, and EE were involved in the conception of the idea, methodology design, performed data analysis and interpretation, and prepared the manuscript for publication and revision. All authors have read and approved the final manuscript.

## Funding

The authors would like to acknowledge the financial support for this work from the Deanship of Scientific Research (DSR), University of Tabuk, Saudi Arabia, under the no. 0131-1442-S.

## Conflict of interest

The authors declare that the research was conducted in the absence of any commercial or financial relationships that could be construed as a potential conflict of interest.

## Publisher's note

All claims expressed in this article are solely those of the authors and do not necessarily represent those of their affiliated organizations, or those of the publisher, the editors and the reviewers. Any product that may be evaluated in this article, or claim that may be made by its manufacturer, is not guaranteed or endorsed by the publisher.
